# P03.12. Use of the electronic medical record to teach complementary and alternative medicine: what's the impact?

**DOI:** 10.1186/1472-6882-12-S1-P265

**Published:** 2012-06-12

**Authors:** R Teets, A Cohrssen, P Jennifer, J Silberlicht

**Affiliations:** 1 Albert Einstein College of Medicine, Bronx, USA; 2 Institute for Family Health, New York City, USA

## Purpose

Although some progress has been made in recent years, training in how to counsel patients effectively on complementary/alternative medicine (CAM) is not widely integrated into undergraduate medical education. Our purpose in this study was to evaluate the impact of a teaching intervention utilizing the electronic medical record (EMR) as a point-of-care learning tool on students’ attitudes and knowledge of CAM and on their self-reported competence in counseling patients on this subject.

## Methods

Students are oriented to the EMR by the preceptor and provided with a one-hour didactic centered on CAM. This lecture orients students to available CAM modalities; discusses evidence-based medicine and CAM; surveys herbs and supplements with supporting evidence; and finishes with a case-study illustrating how CAM can be brought into a patient encounter, using CAM “smartphrases,” which are essentially premade templates readily accessible in the EMR. These smart-phrases provide a real-time reference for students during their patient encounters. Pre- and post- surveys consisting of nine questions using a likert scale examined changes in students’ attitudes about, and use of CAM. These surveys were given anonymously to our medical students. A group of similar medical students who did not rotate here were also given the same surveys, thus representing a control group.

## Results

Among the 27 students in our intervention group, we saw an increase of 48% and 43% in the number of students who felt comfortable counseling patients on fish oil and probiotics, respectively. Among the 38 students in our control group, no significant differences were observed.

## Conclusion

Results of this small sample pilot study suggest that CAM lectures plus EHR-based CAM assessments and tools can increase student comfort with counseling on probiotics and fish oil. The fact that this effect was not seen for acupressure and mind-body interventions suggests a different intervention may be needed.

